# A Low-Profile Dual-Layer Patch Antenna with a Circular Polarizer Consisting of Dual Semicircular Resonators

**DOI:** 10.3390/s18061773

**Published:** 2018-06-01

**Authors:** Li Guo, Ming-Chun Tang, Mei Li

**Affiliations:** College of Communication Engineering, Chongqing University, Chongqing 400044, China; guoli_31@163.com (L.G.); li.mei@cqu.edu.cn (M.L.)

**Keywords:** dual-layer patch antenna, circular polarizer, low-profile, identical frequency response, target detection

## Abstract

In this paper, a circular polarizer comprising dual semicircular split-rings (DSSRs) is presented. By placing it above an elliptical radiator that radiates linearly polarized (LP) waves, dual-layer patch antennas capable of radiating right-hand (RH) or left-hand (LH) circularly polarized (CP) waves are achieved in terms of the different offset direction of the bottom splits of the DSSRs. Because of both the capacitive coupling to the radiator and the degenerate modes existing in the excited DSSRs, the DSSRs collaboratively result in a circularly polarized radiation, successfully converting incident LP waves into CP ones. Simulated results show that the impedance, axial ratio (AR), and gain frequency response of both proposed CP antennas are identical, with a simulated 3-dB AR bandwidth of 72 MHz covering 2.402–2.474 GHz and a gain enhanced by 3.9 dB. The proposed antennas were fabricated and measured, revealing an operational bandwidth of 65 MHz (2.345–2.41 GHz) and a peak gain up to 9 dBi. Moreover, a low profile of 0.063λ_0_ is maintained. The proposed CP antennas could be as a candidate for wireless target detection applications in terms of their identical frequency response property.

## 1. Introduction

Contemporary wireless communications are steering to large capacity, integrated functions, and high reliability. However, multipath reflection and polarization mismatching between the transmitters and receivers make communications that apply LP antennas unreliable [1]. CP antennas can transmit/receive LP waves in an arbitrary direction or sense identical CP waves, which significantly lowers the transmission blocking caused by polarization inconsistency [2]. There are many applications where CP antennas can be used [3,4]. Especially in a wireless target detection field, an electromagnetic wave is usually utilized in a manner that captures the reflected/scattered wave from targets, representatively operated as radars [5]. However, in many situations, the objective/goal may be knowing a target’s state, such as its location or orientation, but it is difficult to obtain the environmental characteristics surrounding targets, such as background or obstacles, which could provide a more comprehensive understanding of targets [6].

**Figure 1:**
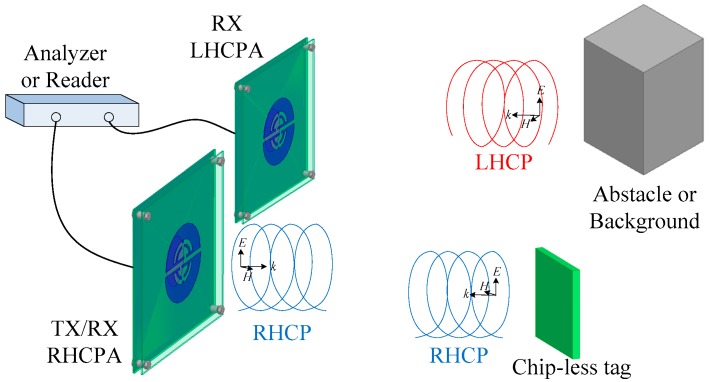
Principle of operation of the target detection based on chip-less tag technology.

There is a feasible target detection solution to resolve the above difficulties, that is, applying chip-less radio-frequency identification (RFID) technology, which has been a topic of growing research interest for several years [7,8]. There exist several frequency bands in RFID applications, e.g., UHF 0.92 GHz [9] and ISM 2.45 GHz [10,11], due to their far-field detection capacity and high data transfer rate. Since common tag antennas have low gain values, a high-gain reader antenna becomes essential for far distance detection [12]. In the chip-less RFID system considered here, the circularly polarized signal is employed for both reader and tag, as shown in Figure 1. To comprehensively detect target information including background and the obstacles around it, an RHCP and an LHCP reader antenna are adopted, which have identical impedance, AR, and gain frequency response for convenient signal processing, and are placed close to each other with low mutual coupling [13]. The main reader antenna is transmitting the RHCP wave and receiving the RHCP wave radiated from tags. The assistant reader antenna is receiving the LHCP wave reflected from the background or obstacles. All the signals are inputting into an analyzer/reader to be extracted and contrasted so as to recognize targets, background, and/or obstacle characteristics. This signal analysis and extraction can be effectively implemented by resorting to the same frequency response property of two reader antennas and the experimental data tested in a previous study [13].

It is critical to properly select the reader and tag antennas, as mentioned previously. Stacking a circular polarizer above microstrip patch antenna can be one design configuration to meet the requirement that two circularly polarized reader antennas possess an identical frequency response [1,14]. This is because the RHCP and the LHCP waves are converted from the same LP source and it is possible to achieve identical CP radiation performance by adjusting or changing the structure of the circular polarizer [15]. In the literature, circular polarizers are commonly designed using structures such as metasurfaces [16], meander lines [17], strips, and resonators [18]. Typically, a certain number of them are based on periodic structures of an anisotropic/isotropic material nature and have been integrated into antenna designs [19,20]. However, for these antennas, the spacing between antennas and polarizers is deemed to be thick. For instance, in the case of integrating a conventional meander-line polarizer, the spacing is nearly a quarter wavelength [17]. In addition, it is usually difficult to make the frequency response of right- and left-hand circular polarization identical to meet the demand of certain dual-sense applications [19]. As a solution, it is easier to use resonators to control or adjust the antenna performance [21]. In particular, if several resonant elements for circular polarization are placed above a microstrip patch antenna that radiates LP waves, the corresponding orthogonal modes would be excited due to the near-field coupling to the bottom radiator and could be tuned individually with subtle changes to the resonant elements. Likewise, these polarizers could be applied in environments demanding temporal polarization transformation, such as for satellite communication [22,23], antenna arrays and radomes [24,25].

In this paper, based on single-fed probe structures, dual-layer, low-profile patch antennas with RHCP and LHCP characteristics were designed. A circular polarizer printed with resonant DSSRs as a superstrate is stacked above an elliptical patch antenna to convert the LP field of an elliptical patch antenna into a CP one. Under the capacitive coupling to the elliptical patch antenna below, the DSSRs collaboratively create a pair of orthogonal modes of approximately equal amplitude and of 90° relative phase shift so that a CP mode is produced. In addition, RHCP and LHCP patch antennas are achieved with identical frequency response by simply changing the offset orientation of the dual bottom splits of the DSSRs. The physical mechanism of the circular polarizer and the antenna configurations are illustrated in Section 2, and the antennas’ impedance, axial ratio, and radiation performance are simulated, measured, and analyzed in Section 3. The simulation tool HFSS version 13.0 is used in this paper.

## 2. Circular Polarizer and Circularly Polarized Patch Antenna Design

### 2.1. Design of Circular Polarizer

**Figure 2:**
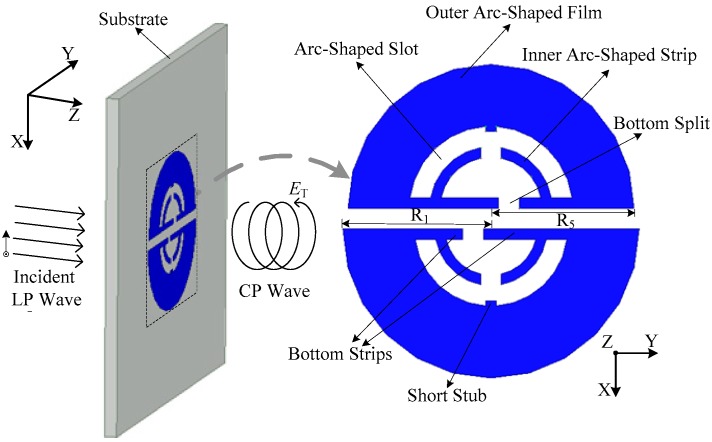
Configuration of the proposed circular polarizer.

A circular polarizer consisting of the DSSRs is investigated. DSSRs mainly consist of two face-to-face placed semicircular split-rings (SSRs) that are printed on the substrate of a Rogers Duroid 4350B (ε_r_ = 3.48, tanδ = 0.0037, with dimensions 132 mm × 132 mm × 1 mm), as shown in Figure 2. The polarizer is excited by a LP incident wave with x-polarization, which is converted into a CP transmitted one as it passes through the polarizer. For the single semicircular split-ring, it was obtained by bending a conventional circular split ring into a semicircular one so that the resulting compact structure could assemble more electromagnetic energy as it was constituted into a circular polarizer. Then, most of the work was directed at the design and optimization of the inner part. Here, one important step is that double bottom strips are horizontally inserted along its diameter edge. Also, a short stub segment is inserted at the top of the inner side of the arc-shaped film for enhancing and tuning the CP radiation. Dual inner arc-shaped strips are separated by a split located between their ends and are connected to the outer arc-shaped film through bottom strips. An arc-shaped slot is formed between them. Also, a bottom split is generated due to the separated bottom strips. The dual SSRs are in a similar structure, as previously illustrated, but mainly differ in their bottom split, which inversely offsets an equal distance from the semicircular center. Moreover, the structures are slightly different in dimensions. In detail, the SSR (with outer radius R_5_) in the upper half-plane is smaller than the one (with R_1_) in the lower half-plane. In addition, the dual SRRs present an approximate elliptical shape on the whole, which can generate orthogonal modes when excited properly and thus result in a circularly polarized radiation [26].

**Figure 3:**
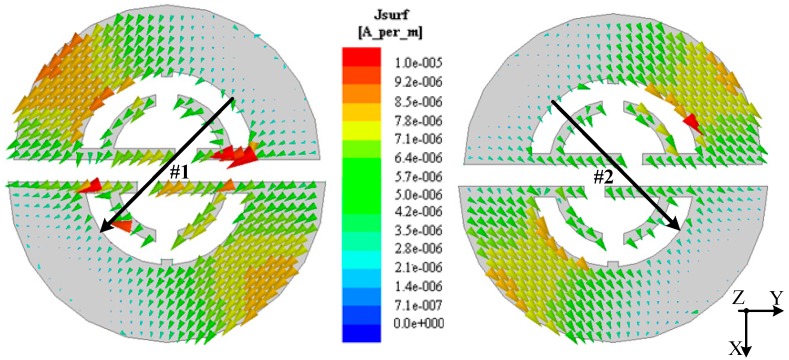
Surface current distribution on the DSSRs of the polarizer at its modes #1 and #2, corresponding eigenfrequencies *f*_#1_ = 2.5189 GHz and *f*_#2_ = 2.5190 GHz, respectively, in terms of the eigenmode model.

According to the circular polarizer proposed above, a cavity model containing it was constructed in HFSS using an eigenmode solution [27]. Then, the surface current distributions of the DSSRs were obtained, as shown in Figure 3. It was found that a pair of degenerate orthogonal modes (mode #1 and #2) with adjacent eigenfrequencies of 2.5189 GHz and 2.5190 GHz, respectively, was generated, related to the orthogonal modes of the CP radiation of the polarizer. Also, it can be seen that the surface currents have a considerable intensity on the edge of the slots and mainly flow on the outer films and the thin inner strips (though relatively small) of DSSRs along the 45° titled direction. This is partly because the outer films and the inner strips contribute to the inductive effect of DSSRs, and the corresponding capacitive ones are mainly attributed to the slots in the eigenmode model, where both result in considerable resonance. Note that while the polarizer integrated into the antennas is considered in this paper, the resulting currents would be primarily distributed on the edge of the slots and the inner strips due to the center-placed elliptical radiator’s excitation making electromagnetic energy accumulate on the inner part of DSSRs. In this case, the outer films still operate for the impedance matching and the inner part plays an important role in achieving the circular polarization and can easily be adjusted to control the CP performance. Also, it can be observed that the surface currents vary in magnitude by close to a half wavelength when flowing along the 45° titled direction of the DSSRs and corresponding to its orthogonal direction, the current is non-uniform due to the outer films and inner strips of different configuration but approximately symmetrical. Alternatively, the current intensity for both modes appears to make a small difference. It can be attributed to the structure difference along the two orthogonal tilted directions of the DSSRs. Since we can derive the radiation field from the surface current on the radiator, the total far-field *E_Z_*_T_ radiating from the polarizer as it integrates into antennas can be expressed as follows [28]:

(1) $$E_{ZT}=k_{\#1}E_{\#1}+k_{\#2}E_{\#2},$$

where *E*_#1_ and *E*_#2_ are the electric field components relative to mode #1 and #2, respectively, and *k*_#1_ and *k*_#2_ are corresponding weight coefficients. The calculation of *k*_#1_ and *k*_#2_ can be carried out by applying weight functions (for example, one possible matrix representation is $\left[\begin{array}{cc} 1 & 1\\ -1 & 1 \end{array}\right]$, which takes into account the interaction between *E*_#1_ and *E*_#2_), by multiplication and integration on both sides of Equation (1). Furthermore, the AR of the radiated EM field can be calculated by using *k*_#1_ and *k*_#2_ in terms of the following equation [28,29]:

(2) $$AR=\sqrt{\frac{{\left|k_{\#1}\right|}^{2}\cos^{2}r+\left|k_{\#1}\right|\left|k_{\#2}\right|\sin2r\cos\Delta \varphi +{\left|k_{\#2}\right|}^{2}\sin^{2}r}{{\left|k_{\#1}\right|}^{2}\sin^{2}r-\left|k_{\#1}\right|\left|k_{\#2}\right|\sin2r\cos\Delta \varphi +{\left|k_{\#2}\right|}^{2}\cos^{2}r}}.$$

In this way, for the polarizer we found that *k*_#1_ = *k*_#2_, |*E*_#1_|≈|*E*_#2_|, and ∠*E*_#2_ − ∠*E*_#1_ = 90° (∠*E*_#2_ = 45°, ∠*E*_#1_ = −45°, which are referring to the +x-axis). Thus, a RHCP polarizer is achieved. In contrast, if we properly change the polarizer and control the parameters such that ∠*E*_#2_ − ∠*E*_#1_ = −90° (∠*E*_#2_ = −45°, ∠*E*_#1_ = 45°), the LHCP characteristics can be achieved. The offset distance of the bottom split can be used to tune the phase difference between *E*_#1_ and *E*_#2_.

**Figure 4:**
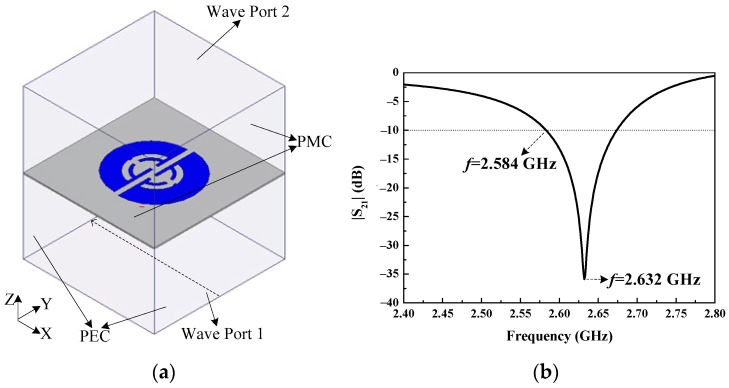
(**a**) Waveguide model for investigation on the proposed polarizer; (**b**) |S_21_|.

**Figure 5:**
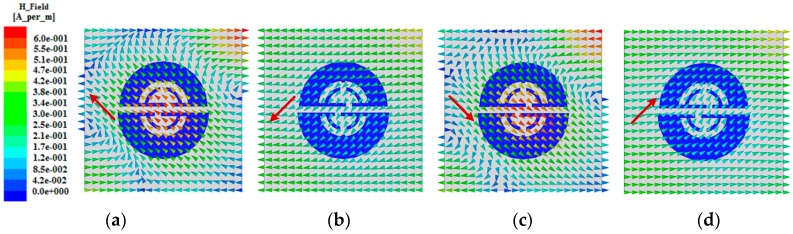
Magnetic field distributions for the polarizer validation from the waveguide model in Figure 4a. The surface magnetic field distributions on the top side of the model at 2.584 GHz are obtained for four different phase intervals: (**a**) 0°; (**b**) 90°; (**c**) 180°; and (**d**) 270°.

Further investigation and validation to the physics of the circular polarizer were carried out in terms of the waveguide model, as shown in Figure 4a. As mentioned previously, the polarizer is illuminated by LP waves below, the bottom side of the waveguide model is set to wave port 1, and the polarizer is suspended above. Also, a perfect electric conductor (PEC) and a perfect magnetic conductor (PMC) boundary are assigned to the model’s opposite sides lying on the x- and y-axis, respectively. Furthermore, wave port 2 is set up on the top side of the model. Thus, as an incident LP plane wave transmitting from wave port 1 and passing through the polarizer, a CP wave would be expected to be found at wave port 2 as the operation implied by circular polarizers. Based on such a model, it was found that revising the offset of the dual bottom splits that go along the opposite direction to each other results in different sensing circular polarizations. Also, with a proper equal offset distance assigned to the dual bottom splits, such as 3.5 mm (along the direction as the bottom splits shown in Figure 4a), a considerable CP (RHCP) property can be achieved. In fact, we can fix the offset distance in the 3.5 ± 0.5 mm range, where an acceptable CP and impedance matching performance can be maintained. While revising the dual bottom splits at such an equal distance, opposite sensing CP (LHCP) characteristics can be obtained. With appropriate separations between the polarizer surface and the model’s upper and lower sides maintained, and a carefully optimized substrate size of the polarizer, the transmission coefficient |S_21_| between the wave port 1 and 2 was calculated as shown in Figure 4b. A resonance centered at 2.632 GHz can be seen from this figure, which is associated with the circular polarization. Additionally, for the RHCP case the surface magnetic field distributions on the top side of the model (i.e., port 2) at 2.584 GHz within a 10-dB range are investigated and shown in Figure 5a–d for four different phases: 0°, 90°, 180°, and 270°, respectively. One can observe that an RHCP characteristic appears as the tip of the magnetic vectors change counterclockwise within one period, which verifies the polarizer’s operation. For the LHCP case, the corresponding |S_21_| and magnetic field distributions at the four phases are similar to the RHCP case, except for the magnetic vectors varying clockwise. Since the polarizer at the RHCP case is a mirror image of itself at the LHCP case, the performance of the constituted antenna in RHCP and LHCP should be identical [15]. In addition, the utilized waveguide model has two PEC sides of opposite and a large plane source is imposed on port 1, which could result in the incident field (used for excitation of the polarizer) having characteristics approximate to the near field from the source. Thus, when the polarizer is integrated into a microstrip antenna and excited by the EM in near field, it could have similar characteristics to the polarizer placed in the environment of Figure 4a.

### 2.2. Circularly Polarized Patch Antennas Design

**Figure 6:**
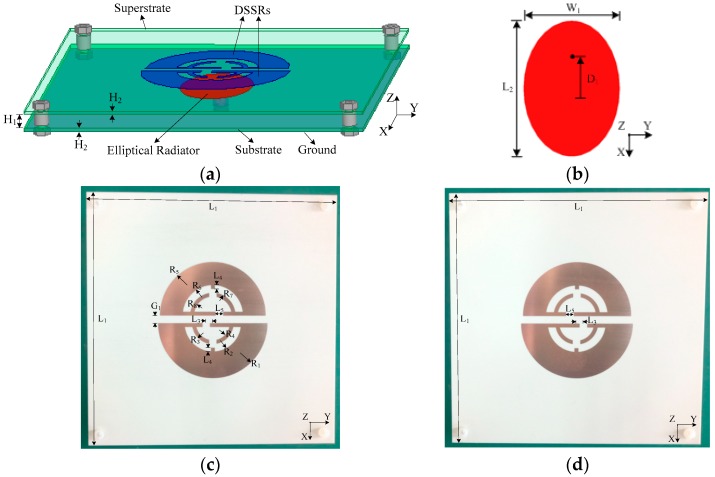
Configuration of the proposed circularly polarized antennas: (**a**) Three-dimensional view; (**b**) elliptical radiator; (**c**,**d**) are, respectively, the front view of fabrication prototype corresponding to the RHCPA and the LHCPA.

The right-hand circularly polarized patch antenna (RHCPA) was constructed with the circular polarizer illustrated above and an elliptical patch antenna (EPA), mainly consisting of an elliptical radiator with a ground placed on the bottom side of the substrate. They are in a stacked arrangement and separated by an air gap. The detailed configurations of the RHCPA and the LHCPA are shown in Figure 6a–d. Note that the dual bottom splits are offset from the center at a distance of L_3_ = 3.5 mm. A probe-fed manner is employed with the feeding point located on the long axial of the elliptical radiator and offset from its center so that an LP wave can be radiated from the EPA. The DSSRs are coupled to be resonated in the near field of the driven patch (EPA), and they collaboratively create orthogonal modes of approximately equal amplitude and a relative phase shift of 90° for CP characteristics. Also, the strong coupling with the EPA brings a low profile property for the proposed antennas. In addition, four Teflon screws are mounted at the four corners of the substrates to support the polarizer. The dimensions associated with the configurations were optimized for RHCP characteristics, and are listed in Table 1. It was worth mentioning that the dimensions (such as semicircular radius R_1_ and R_5_ and elliptic axis length L_2_ and W_1_) could be easily scaled to fit other frequency band applications, such as UHF and GPS band.

As with the circular polarizer analyzed previously, reversing the offset of the dual bottom splits of the DSSRs leads to the other sensing circular polarization. Hence, an alternative configuration of DSSRs with the proposed antennas was considered, as shown in Figure 6d, for which the dual bottom splits are reversed at equal offset distance of L_3_ = 3.5 mm. Thus, a second proposed antenna with such DSSRs keeping the same EPA and superstrate slab dimensions as the RHCPA was obtained and termed left-hand circularly polarized patch antenna (LHCPA). The corresponding characteristics will be demonstrated as follows.

## 3. Simulations and Experiments

The proposed circularly polarized antennas were constructed and simulated with high-frequency simulation software (HFSS). According to the EPA, RHCPA, and LHCPA discussed previously, the parameters of these antennas, such as reflection coefficient (|S_11_|), axial ratio, and radiation pattern, were calculated and analyzed. Furthermore, the measured reflection coefficient was obtained by a vector network analyzer (VNA), and the radiation performance was measured in a multi-probe spherical near-field test anechoic chamber.

**Figure 7:**
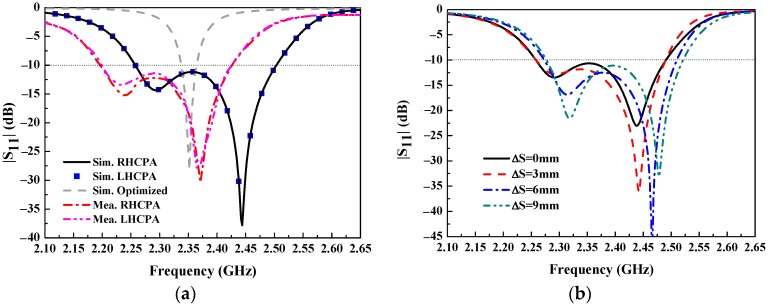
(**a**) Simulated and measured reflection coefficients of the optimized EPA, the RHCPA, and the LHCPA; (**b**) effect of inversely offsetting SSRs by equal distance (ΔS) on the reflection coefficients of the RHCPA.

Simulated and measured |S_11_| of the optimized EPA (without the polarizer), the LHCPA, and the RHCPA are shown in Figure 7, in which the simulated |S_11_| of the optimized EPA was obtained by optimizing the feed position of the EPA. It can be observed that the impedance bandwidth (where |S_11_| ≤ −10 dB) of the optimized EPA is 26 MHz (fractional bandwidth 1.1%, 2.338–2.364 GHz) with respect to the center frequency 2.352 GHz. One can observe that the |S_11_| curves of the RHCPA and the LHCPA are overlapped over the whole considered frequency band, with an identical impedance bandwidth of 244 MHz (fractional bandwidth 10.2%, 2.258–2.502 GHz), even though their bottom splits are in opposite offsetting, which validates the identical performance predicted previously. While the polarizer is stacked above the EPA, the impedance bandwidth is widened nearly 9-fold due to the overlapping of the lower and upper resonant dips, which are attributed to the polarizer and the EPA, respectively. The measured impedance bandwidth of the RHCPA and the LHCPA are 240 MHz (fractional bandwidth 10.3%, 2.19–2.43 GHz). However, their curves are not completely overlapping over the frequency band as with the simulation case, though they are very close to each other. This is mainly due to the fabrication difference between the RHCPA and the LHCPA. It can be seen that there is about a 70 MHz downward frequency shift in the measured results compared to the simulated ones. This can be attributed to the practical dielectric loss, which makes the effective dielectric constant increase [30,31]. It is worth mentioning that one can select a large dielectric board/plane size for the proposed antennas to obtain a higher gain. Figure 7b shows |S_11_| varying with the inverse offsetting of two SSRs of the RHCPA: the small SSR shifts to the right while the large one shifts to the left by the same offsetting distance (ΔS). This indicates that the covered frequency band can be slightly adjusted under a nearly unaltered bandwidth. Otherwise, the fixed longitudinal spacing between SSRs brings an appropriate coupling to maintain the resonance. In addition, when putting the prototypes of the RHCPA and the LHCPA side by side, the transmission coefficient |S_21_| between the two ports of both antennas was less than −33 dB, which indicates that the coupling between the two antennas is very small [13].

**Figure 8:**
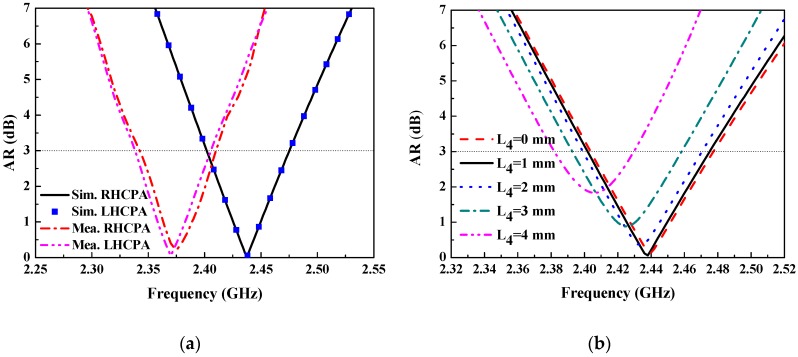
(**a**) Simulated and measured AR of the RHCPA and the LHCPA versus frequency; (**b**) AR optimization with varied short stub length corresponding to the RHCPA.

Simulated and measured AR in the broadside (along +z axis) of the RHCPA and the LHCPA are shown in Figure 8a. It can be seen that their simulated AR curves overlapped in the entire band once again, with the same 3-dB AR bandwidth 72 MHz (fractional bandwidth 2.95%, 2.402–2.474 GHz), and the minimum AR value of 0.059 dB is observed at frequency 2.438 GHz. For the measured results, the curves are not overlapping due to fabrication error. The 3-dB AR bandwidth for the LHCPA and the RHCPA covers 2.345–2.41 GHz (fractional bandwidth 2.7%) and 2.34–2.405 GHz (fractional bandwidth 2.7%), respectively, with a minimum AR value of 0.05 dB at 2.37 GHz. In the same manner, a frequency shift is observed for both antennas. Nevertheless, all the AR bandwidths are within their impedance bandwidth. The low AR value was obtained with a substantial optimization process, resorting to the inserted short stubs and optimization of their length L_4_. Figure 8b shows a set of AR curves with respect to frequency with varied L_4_. It is found that the AR has the minimal value at L_4_ = 1 mm, which is in the intermediate range of L_4_ considered. Otherwise, the 3-dB AR bandwidth can be attained in a proper frequency range. However, with L_4_ deviating from the optimum value, the AR deteriorates and the AR bandwidth becomes narrower. Actually, these deteriorations can be alleviated by further tuning other structures of the DSSRs so as to realize good AR performance.

**Figure 9:**
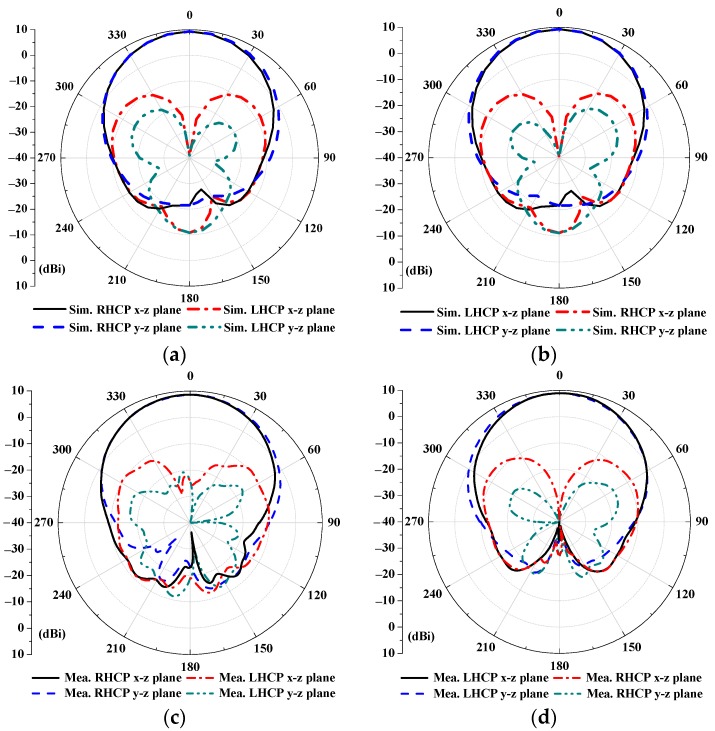
Radiation patterns of (**a**) RHCPA and (**b**) LHCPA in simulation; and (**c**) RHCPA and (**d**) LHCPA in measurement, at frequency 2.438 GHz and 2.37 GHz, respectively.

Simulated and measured two-dimensional radiation patterns of the RHCPA and the LHCPA are shown in Figure 9a–d. The realized gain and the cross-polarization of the RHCPA and the LHCPA are, respectively, 9.25 dBi and lower than −10.9 dBi at 2.438 GHz, as seen from Figure 9a,b. For the measured results, Figure 9c,d show a gain of 8.54 and 8.86 dBi at 2.37 GHz, with the cross-polarization lower than −9.71 and −10.52 dBi for both antennas, respectively. In addition, the measured (simulated) half-power beamwidths for both antennas are approximately 60° across their 3-dB AR bandwidth. As indicated from these results, the conversion from the linear polarization to the right-hand or the left-hand circular polarization is verified after adding their respective polarizers. In addition, after reversing only the offset direction of the dual bottom splits of the DSSRs, the corresponding RHCP and LHCP characteristics of the proposed antennas are validated.

**Figure 10:**
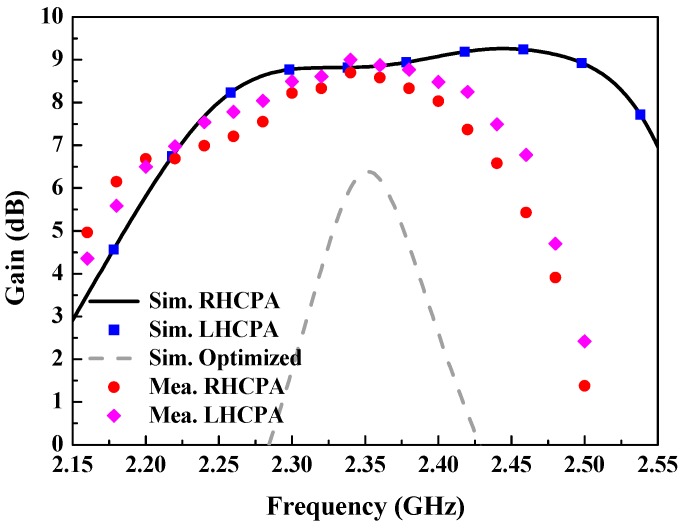
Simulated and measured gain of the RHCPA and the LHCPA and simulated gain of the optimized EPA as a function of frequency.

Simulated and measured gains in the broadside direction of the RHCPA and the LHCPA are plotted in Figure 10. Both simulated curves coincide and have the same peak gain up to 9.26 dBi, which is increased by about 3.9 dB compared to that of the optimized EPA. Regarding the measured results, the peak gains of both antennas are up to 8.7 and 9 dBi, respectively, with a variation of less than 0.7 dB across their 3-dB AR bands. The acceptable discrepancy between simulation and measurement can be ascribed to fabrication and measurement tolerances. In addition, the simulated efficiency of the RHCPA and the LHCPA is higher than 89% over their 3-dB AR bands. Table 2 compares the performance of the proposed circularly polarized microstrip antennas with that of two other antennas reported in the recent literature. It can be observed that the patch antennas proposed in this paper exhibits superiority in CP bandwidth, profile height and gain. In particular, their profile height of 0.063λ_0_ is approximately one-half of the contrasted lowest one (0.13λ_0_) while maintaining a wider CP bandwidth and higher gain, which manifests their low profile characteristics. It should be mentioned that, in our previous work [23] the CP antenna was built in a multi-layer patch structure without air gap, this has a lower profile height (0.016λ_0_). In contrast, in the present work our aim is to achieve higher antenna gain and radiation efficiency by inserting an air-gap layer for applying to remote RFID systems.

## 4. Conclusions

This paper presents a dual-layer CP patch antenna with a circular polarizer that contributes to the polarization transformation from an LP wave to a CP one. The circular polarizer mainly consists of DSSRs with opposite-offset bottom splits. By simply reversing the offset orientation of the dual bottom splits of the DSSRs, the RHCPA and the LHCPA can be realized with the same |S_11_| and AR frequency response, only differing in radiation pattern, namely corresponding to the RHCP and LHCP radiation patterns, respectively. The measured 3-dB AR bandwidth of the proposed LHCP antennas is 65 MHz (fractional bandwidth 2.7%), covering 2.345–2.41 GHz, and its peak gain is as high as 9 dBi. The frequency shift between simulation and measurement is mainly owing to the practical dielectric loss leading to a rising effective dielectric constant. Dual-sensing CP characteristics are attributed to the structural symmetry for flexibly changing the orthogonal modes of the DSSRs that arise from the capacitive coupling resonance. The proposed antennas are kept with an overall size of 1.04λ_0_ × 1.04λ_0_ × 0.063λ_0_, and their performance characteristics could be suitable for wireless target detection applications.

## Figures and Tables

**Table 1:** The dimensions of the proposed antennas shown in Figure 6 (unit: mm).

**Parameters**	L_1_	L_2_	L_3_	L_4_	L_5_	W_1_	W_2_	H_1_	H_2_	H_3_
**Value**	132	39.6	3.5	1	4	28	2	5.5	1	1.5
**Parameters**	R_1_	R_2_	R_3_	R_4_	R_5_	R_6_	R_7_	R_8_	D_1_	G_1_
**Value**	29	15	11	9	28	16	12	10	9.6	4

**Table 2:** Comparison between the performance of the proposed circularly polarized microstrip antennas and a few other reported antennas with circular polarizers.

Ref. Work	[20]	[25]	Present Work
**3-dB AR Fractional B.W. (%)**	2.55	2.1	2.7
**Gain (dBi)**	11	8.2	9
**Profile height of antennas (λ_0_)**	0.43	0.13	0.063
**CP frequency range (GHz)**	13.55–13.9	1.547–1.58	2.34–2.405
